# Breath Analysis via Surface Enhanced Raman Spectroscopy

**DOI:** 10.1021/acssensors.4c02685

**Published:** 2025-01-17

**Authors:** Adrián Fernández-Lodeiro, Marios Constantinou, Christoforos Panteli, Agapios Agapiou, Chrysafis Andreou

**Affiliations:** † Department of Electrical and Computer Engineering, University of Cyprus, Nicosia 2112 Cyprus; ‡ Department of Chemistry, University of Cyprus, Nicosia 2112, Cyprus

**Keywords:** Surface-Enhanced Raman Spectroscopy (SERS), Breath, Sensors, Nanomaterials, Volatile Organic Compounds (VOCs), Machine Learning, Preconcentrators, Microfluidics

## Abstract

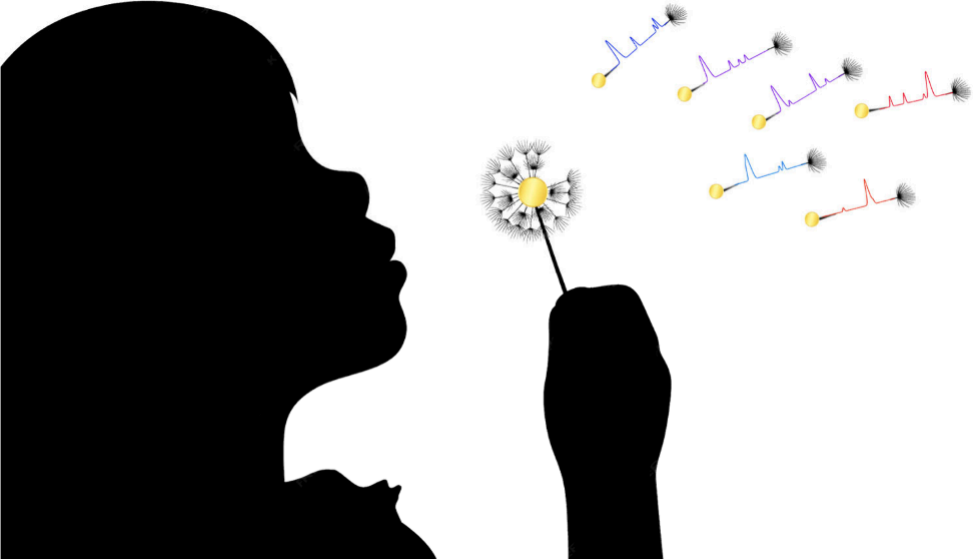

Breath analysis is increasingly recognized as a powerful noninvasive diagnostic technique, and a plethora of exhaled volatile biomarkers have been associated with various diseases. However, traditional analytical methodologies are not amenable to high-throughput diagnostic applications at the point of need. An optical spectroscopic technique, surface-enhanced Raman spectroscopy (SERS), mostly used in the research setting for liquid sample analysis, has recently been applied to breath-based diagnostics. This promising noninvasive diagnostic tool has been demonstrated for the identification of various diseases, including lung cancer, gastric cancer, and diabetes. The versatility of SERS has enabled the use of different diagnostic strategies and allowed for fast and accurate detection of small analytes in exhaled breath. In this review, we provide an overview of recent advances in SERS-based breath analysis, focusing on sensors for the detection of gases and volatile organic compounds (VOCs) in exhaled breath, and highlight generic strategies for sample preconcentration and methods for spectral analysis. We aim to provide an overview of the state of the art and inspiration for further SERS investigation of expiration.

With approximately 20,000 breaths taken by every human each day, it is clear why breath analysis is one of the easiest and most accessible methods for assessing a person’s health. The first attempts to identify the importance of breath date to ancient times, when the Greek physician Hippocrates (c. 460–370 BCE) believed that diseases were caused by the formation of harmful vapors arising from poorly digested food residues.^1^ With every exhaled breath, molecules indicative of one’s health status are expelled; gases like nitric oxide (NO) and hydrogen sulfide (H_2_S) have been used as diagnostic markers, but the most commonly reported diagnostic molecules are volatile organic compounds (VOCs). VOCs are organic chemicals with a high vapor pressure at room temperature, allowing them to evaporate into a gas state.^2^ VOCs from different sources can be found in various environments, including indoor^3^ and outdoor air,^4^ water,^5^ and soil.^6^ They are produced commonly from biological processes, but they can also be released through various other means, including industrial activities,^7^ vehicle emissions,^8^ and natural nonbiological sources.^9^ Environmental VOCs can contribute to the formation of ground-level ozone and particulate matter, which can exacerbate respiratory problems and other health issues^10^ making their detection crucial due to their potential impact on human health.^11^

VOCs are released from the body through various means, including through the skin,^12^ urine,^13^ feces,^14^ or vaginal secretions.^15^ Among them, the analysis of trace VOCs emitted via breath has gained significant attention due to its potential as a noninvasive, rapid, and accurate diagnostic tool for various diseases and health conditions.^16^ The molecular composition of breath can be influenced by various factors, including intrinsic metabolic processes,^17^ food and medication, as well as environmental exposure.^18^ The analysis of VOCs in breath has been recently explored to diagnose various diseases, including lung cancer,^19^ diabetes,^20^ and gastrointestinal disorders,^21^ among others. The importance of VOC analysis in breath lies in its potential impact on the field of diagnostics and personalized medicine. VOC analysis has seen significant advancements, with the evolution of new methods and technologies: the field now employs various sophisticated techniques for sample preconcentration, including needle trap devices (NTDs)^22^ and solid-phase microextraction (SPME),^23^ followed by exquisite analytical identification of the VOCs using gas chromatography–mass spectrometry (GC-MS),^24^ single gas sensors, laser spectroscopy,^25^ or emerging technologies based on the concept of an electronic nose.^26^ Analysis of breath offers several advantages over traditional diagnostic methods, including noninvasiveness and potentially low cost, depending on the analytical method. It can provide real-time information about an individual’s health status, allowing for early detection of diseases and timely intervention.^27^

Recent research has investigated the feasibility of using nanostructured sensors based on surface-enhanced Raman scattering (SERS) for detecting VOCs. This approach leverages the vibrational fingerprints of molecular structures, which provide distinct Raman peaks to identify the different analytes. One of the first reports for SERS-based VOC detection around the turn of the century was not focused on breath. The study utilized roughened silver substrates and thermoelectric cooler-based SERS systems to detect VOC vapors like trichloroethylene (TCE);^28^ this strategy, consisting of a preconcentration step and subsequent detection, has proven effective and has been followed by subsequent works. With recent advancements, methodologies have significantly improved, with demonstrations of multiplex SERS detection for VOCs and substrates with high sensitivity.^29−32^ This has enabled the successful detection of multiple VOCs with a single sensor, including some with low Raman cross sections, such as acetone and ethanol vapors.^29^ In addition to preconcentration and detection, spectral analysis via chemometrics has been recognized as an important component in VOC analysis. Chemometrics involves the application of various statistical, mathematical, and machine learning (ML) techniques to extract meaningful information from chemical measurements. Popular chemometric methods like partial least squares (PLS) and principal component analysis (PCA) are used to differentiate between VOCs in complex mixtures, while more advanced ML algorithms are applied to improve VOC identification and increase the diagnostic value of the detectors.

At the same time, the necessity of sample manipulation and automation has arisen to enable effective sample collection and transport to the sensor, leading to the development of microfluidic devices to support SERS-based sensors. Microfluidics involves manipulating and controlling fluids at the microscale and has been integrated with VOC analysis techniques to enhance the sensitivity and selectivity. Microfluidic systems were shown to improve the sensitivity of detection methods such as SERS by preconcentrating the analytes and mixing them with the buffers and reagents needed for detection. Additionally, microfluidic devices can efficiently handle small volumes of breath samples, allowing for sampling efficacy and automation.

This review highlights promising novel approaches for gas and VOC detection in breath analysis using a variety of SERS substrates. We explore various biomarkers in breath, SERS sensor configurations, preconcentration strategies, and spectral signal analysis methods. SERS-based breath analysis has potential for rapid and effective diagnostic and screening applications.

## Breath Composition and Biomarkers

### VOCs in Breath

In 1971, Linus Pauling was able to quantitatively determine about 250 substances in a sample of breath using gas chromatography.^33^ Since then, breath, consisting of organic and inorganic gases, water vapor, and tiny particles that form aerosols, has served as a vital indicator of overall health. VOCs present in breath can be classified into exogenous or endogenous based on their origin. Exogenous VOCs are introduced into the body through external sources, such as exposure to environmental pollutants, consumption of food, drinks, and drugs, or absorption through the skin. A familiar example of detecting exogeneous VOCs is the breath alcohol test (Breathalyzer), commonly used to determine one’s level of inebriation. On the other hand, endogenous VOCs originate from metabolic processes within the body. These VOCs are produced by cells and tissues during physiological activities such as cellular respiration, digestion, and the breakdown of proteins and carbohydrates. Disease states, abnormal metabolism, and infections also contribute to endogenous VOCs, which may be diagnostically exploited. Accurate analysis of these compounds holds significant potential for the early detection and treatment of conditions such as infections, cancers, and metabolic disorders. More than 200 VOCs were observed in most breath samples, and more than 3000 VOCs were observed at least once, indicating the enormous potential breath analysis holds.^34^ One of the most commonly analyzed VOCs, acetone,^35^ has been identified as a biomarker for diabetes mellitus and is used to monitor ketosis.^36^ It has also been identified as a byproduct of fat metabolism that correlates with the rate of fat loss in healthy individuals.^37^ Aldehydes may be indicative of lung cancer.^38^ Nonorganic volatile molecules have also been used for health assessment; for example, NO is often monitored in patients with asthma.^39^ High ammonia levels in breath can indicate liver dysfunction, kidney disease, and some types of infections.^40^ The detection of H_2_S gas is significant, as abnormal levels are associated with oral diseases, such as periodontitis and halitosis. More recently, the analysis of different carbonyl compounds has proved helpful for COVID-19 detection,^41^ showing the ability of breath analysis to be studied and adapted for a wide variety of health-related problems. A comprehensive list of VOCs may be accessed through the Human Breathomics Database.^42^

### Established Analytical Methods

#### Gas Chromatography–Mass Spectrometry

GC-MS plays a crucial role in analyzing breath samples and is considered the “gold standard”. GC first separates complex mixtures of VOCs into individual components based on their interactions with a stationary phase within a chromatographic column. This separation is essential for isolating the hundreds of diverse compounds in breath. Following separation, MS identifies these components based on their mass-to-charge ratios. This step provides detailed insights into each compound’s molecular structure and concentration, enabling precise identification and measurement. The combination of GC with MS and various preconcentration techniques has been extensively utilized in breath analysis, enhancing the accuracy and sensitivity of detecting and quantifying VOCs in exhaled breath.^23,43^

#### Proton Transfer Reaction–Mass Spectrometry (PTR-MS) and Selected-Ion Flow Tube-Mass Spectrometry (SIFT-MS)

PTR-MS and SIFT-MS are powerful analytical methods for analyzing breath samples. PTR-time of flight (TOF) is highly regarded for its real-time results and high mass resolving power. It operates by ionizing VOCs using proton-transfer reaction ionization, allowing for immediate and direct measurement of trace compounds in breath without requiring sample preparation. This capability makes it well-suited for analyzing breath and provides highly sensitive detection with limits in the sub-parts-per-billion (ppb) range. Additionally, PTR-TOF can measure a complete mass spectrum within a fraction of a second, enabling the identification of chemical compositions and the separation of isobaric molecules.^44^

SIFT-MS is particularly valued for its real-time results and quantitative analysis. It operates by ionizing VOCs with selected precursor ions, allowing for immediate and direct measurement of trace compounds in breath without requiring sample preparation. This capability makes it well-suited for analyzing breath samples and provides highly sensitive detection with limits in the parts-per-trillion range.^45^ On the other hand, TOF-MS offers a high-resolution mass analysis of breath VOCs. It provides precise mass measurements and rapid spectral acquisition, making it ideal for comprehensive untargeted analysis. When coupled with comprehensive 2D gas chromatography (GC × GC), TOF-MS enhances the separation and identification of complex mixtures.^46^

#### Ion Mobility Spectrometry (IMS)

This technique stands out for its high sensitivity, detecting compounds in the parts-per-trillion range. IMS combines high sensitivity and relatively low technical expenditure with high-speed data acquisition. The ability to analyze single-breath exhalations in real time further enhances its utility.^47^

#### Electrochemical Sensors

Electrochemical sensors are prized for their high sensitivity, detection limits reaching the ppb range, and rapid analysis time.^48^ Electrochemical sensors are relatively low-cost and can be miniaturized, making them a versatile option for various applications. Electrochemical sensors have shown great potential in breath analysis via the detection of various compounds. These sensors often use metal oxide semiconductors or conducting polymers as sensing materials, and advancements have included the integration of nanomaterials to enhance sensitivity and selectivity.^49^ Researchers are also exploring novel sensing materials, such as integrating room temperature ionic liquids, to enhance sensor performance.^50^

However, these techniques present some drawbacks. Methods based on MS require specialized equipment, precise calibration, and regular maintenance to ensure valid results. Additionally, data processing is complex and requires trained interpretation.^51^ As a result, the use of MS in clinical settings is limited. The dependence on preconcentrators further complicates its accessibility, particularly in resource-constrained environments, making it less accessible for routine diagnostics compared to more streamlined techniques. Conversely, electrochemical sensors present a different set of challenges in VOC analysis, such as ensuring sufficient selectivity in complex breath mixtures, managing humidity and temperature effects, standardizing sampling procedures, and accurately distinguishing between endogenous and exogenous VOCs.

In this context, a promising, powerful optical spectroscopy technique with different preclinical applications,^52^ SERS, has emerged for breath analysis. SERS offers the possibility of performing cost-effective and quick analysis and, once validated, could produce a robust sensor platform for breath diagnostics. Additionally, SERS offers the possibility of online real-time breath analysis over the offline methodologies used mainly today. Online analysis obviates the need for sample storage and transportation and allows continuous monitoring in a clinical setting, where fast and accurate results are essential. The integration of portable devices, such as hand-held Raman spectrometers and microfluidic sampling systems, could accelerate the adoption of this promising technique, with significant implications for breath-based diagnostics.

## SERS for Breath Analysis

### SERS Fundamental Principles

**Figure 1:**
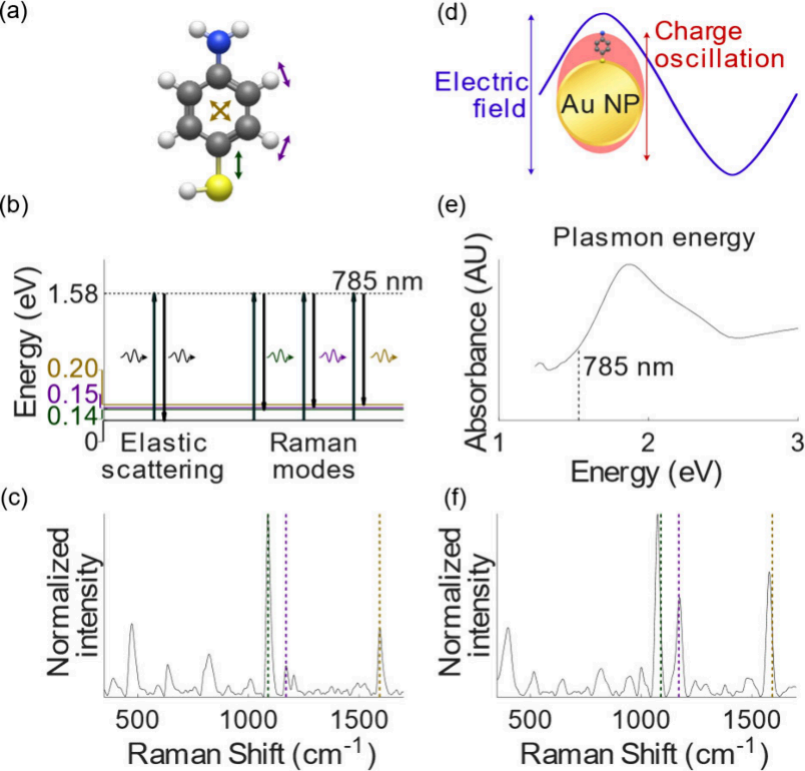
(a) Bond vibrations of molecules, such as in 4-aminothiophenol (4-ATP) shown, have distinct energies. (b) Incident laser light at 785 nm excites these vibrations, producing energy-shifted Raman scattered photons. (c) Raman spectrum of 4-ATP shows the different vibrational energies as characteristic peaks. (d) Collective charge oscillations, called plasmons, excited by the laser on gold nanostructures enhance the local electric field. (e) Plasmon resonance depends on the nanostructure and can be tuned to match the laser energy. (f) SERS spectrum of 4-ATP: the characteristic energies are slightly shifted and greatly enhanced due to bond interactions with the nanoparticle substrate and plasmon, respectively.

When light shines on a material, it typically undergoes elastic scattering. However, a small portion of the photons interact with molecular bond vibrations, exchanging energy and resulting in a wavelength shift in the scattered photon. This inelastic scattering, known as Raman scattering (or the Raman effect), depends directly on the bond vibrational modes and offers a unique spectral fingerprint of the material’s molecular structure (Figure 1a–c). However, this conventional Raman scattering is a rare phenomenon with an exceptionally low probability of occurrence (∼1 in 10^8^ photons).^53^ The Raman effect becomes massively amplified in the vicinity of plasmonic nanostructures, yielding the technique we call SERS (Figure 1d–f). The presence of conductive nanoparticles, or other nanoscale features, amplifies the Raman signal by a factor of 10^10^ or more, enabling even single-molecule detection.^54^ Metal nanostructures enhance Raman signals through two main mechanisms, termed electromagnetic and chemical enhancements, that combine synergistically. The electromagnetic enhancement stems from light interacting with metal nanostructures, typically gold (Au) or silver (Ag), to excite localized surface plasmon resonance (LSPR), which amplifies the local electric field. This excitation significantly boosts the electromagnetic field near the nanostructure, causing molecules adsorbed or near the nanoparticle surface to produce greatly amplified Raman signals. The second mechanism, chemical enhancement, occurs when molecules interact with the metal surface through chemical bonding, along with charge transfer between the metal substrate and the molecule, again enhancing the Raman signal. The sensitivity and effectiveness of SERS depend on the precise control over the size, shape, and arrangement of metal nanostructures and the affinity of the analyte molecule to the substrate.^55^ Forming “hotspots” with intense electromagnetic fields is crucial for maximizing signal enhancement, and this is most easily achieved via nanoparticle aggregation. The hotspot formation depends on the nanoparticles’ morphology and spatial arrangement, critical factors in optimizing plasmonic enhancement.^56^ The great amplification of the Raman scattering cross section allows for the detection of trace amounts of substances, making SERS an exceptionally sensitive analytical technique and a valuable tool for characterizing and detecting trace analytes.

Recent advances in plasmonic sensing have concentrated on developing functional nanostructured materials such as assemblies of metallic nanoparticles to enhance SERS performance. These innovations have expanded the applications of SERS into areas such as chemical analysis, biomedical research, and environmental monitoring. Techniques such as modifying metal nanoparticles with metal–organic frameworks (MOFs), applying silica coatings, or integrating them into hydrogels are among the methods used to improve the signal stability and sensitivity of SERS substrates.

SERS has become a powerful analytical technique, attracting significant attention for detecting and analyzing VOCs since the early 21st century. SERS has been applied across various fields, including environmental monitoring,^57^ drug detection,^58^ and food safety analysis.^59^ However, its potential in biomedical applications has sparked particular interest. The ability to use SERS as a simple, noninvasive, and rapid diagnostic tool has captivated the scientific community seeking to develop point-of-care diagnostics. SERS has been employed for applications from cancer imaging^60^ to detecting biomarkers in breath,^61^ urine,^62^ or blood samples,^63^ offering a noninvasive and swift diagnostic method for various diseases. This technique provides several advantages, including high sensitivity, rapid detection, and the capacity to analyze complex mixtures. However, its usefulness depends on the nanostructured substrates employed. The following subsections present different SERS substrates and surface modifications reported for breath sensing.

### Silver-Based Sensors

Silver-based nanomaterials are widely recognized for their critical role in advancing SERS due to their exceptional plasmonic properties. Among noble metals, silver is particularly valued for its ability to generate strong LSPR, which substantially enhances the Raman scattering signal. This makes silver nanostructures (such as nanospheres, nanowires, and nanocubes) highly effective for SERS-based applications. While silver nanomaterials are prone to oxidation and may exhibit limited long-term stability compared to gold, their superior plasmonic performance and cost-effectiveness make them a preferred choice for many SERS platforms. The ongoing optimization of Ag-based substrates focuses on improving stability while maintaining their high enhancement factors, ensuring their continued relevance in sensitive molecular detection.

**Figure 2:**
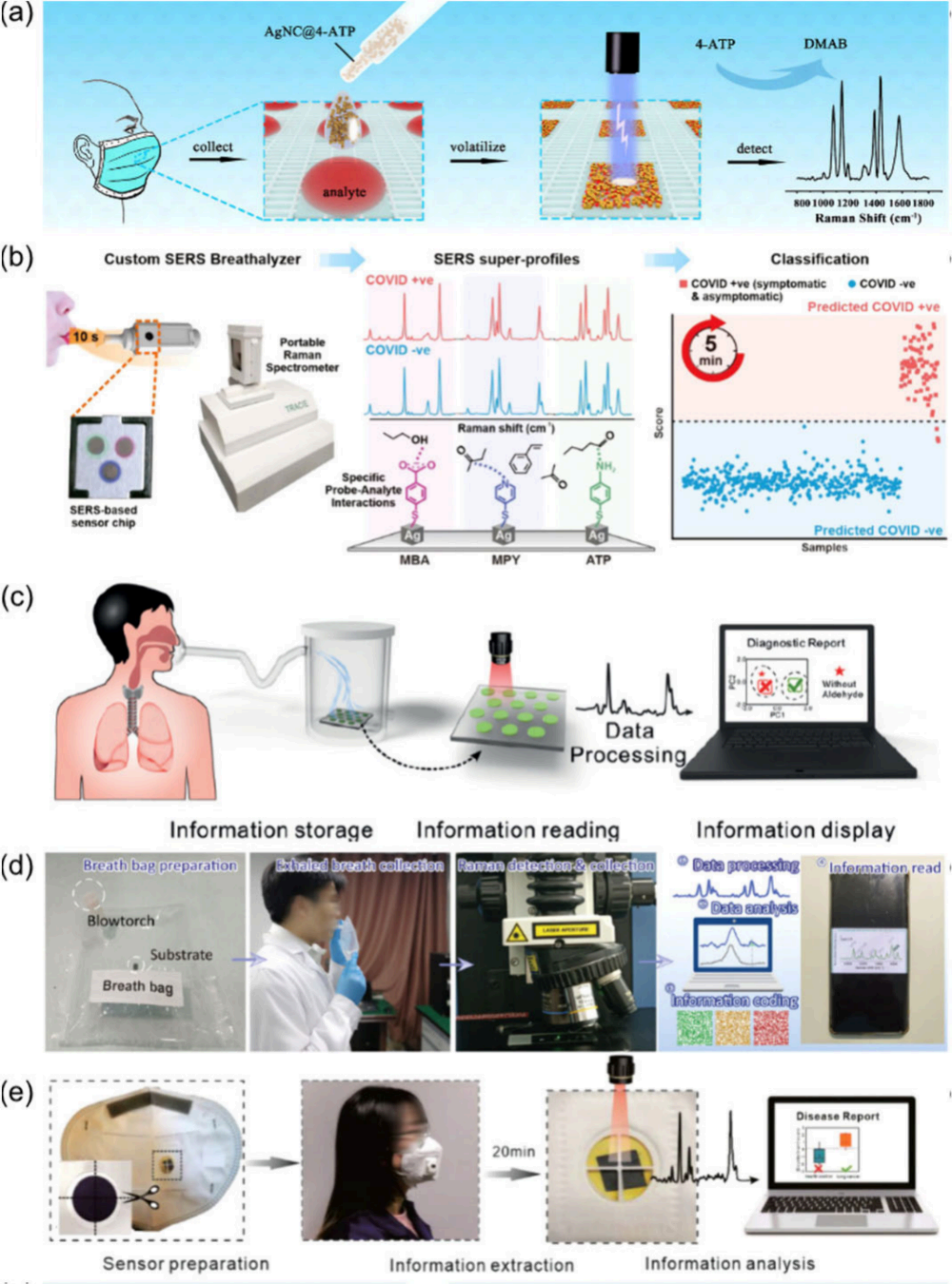
(a) Schematic representation of analyte detection using a mask for collection, AgNC@4-ATP as SERS sensors, and Raman spectroscopy for detection, highlighting the Raman shift peaks for 4-ATP and DMAB. Adapted with permission from ref (64). Copyright 2024 American Chemical Society. (b) Rapid COVID-19 detection using a SERS breathalyzer. From left to right: A person breathes into a custom SERS breathalyzer, capturing the breath sample on a SERS-based sensor chip. The sample is then analyzed using a portable Raman spectrometer. The spectrometer generates SERS superprofiles, showing distinct Raman shifts for COVID-positive and COVID-negative samples, based on specific probe–analyte interactions with the different molecules. The data is classified within 5 min, enabling quick and efficient diagnosis. Adapted with permission from ref (65). Copyright 2022 American Chemical Society. (c) The diagnostic process involves collecting a breath sample (Information Storage), analyzing it with a sensor (Information Reading), processing the data to identify biomarkers (Data Processing), and displaying the results on a computer for diagnosis (Information Display). Adapted with permission from ref (74). Copyright 2019 WILEY-VCH Verlag GmbH *&* Co. KGaA, Weinheim. (d) Steps in the exhaled breath analysis process from left to right: Preparation of the breath bag using a blowtorch and substrate, collection of exhaled breath into the prepared bag, and detection and collection of Raman signals from the breath sample. Processing and analysis of the Raman data, including information encoding, and reading and displaying the processed information on a mobile device. Adapted with permission from ref (77). Copyright 2023 WILEY-VCH Verlag GmbH *&* Co. KGaA, Weinheim. (e) The process of disease detection using a sensor-integrated face mask involves sensor preparation, data collection over 20 min, signal analysis, and generating a disease report. Adapted with permission from ref (80). Copyright 2022 WILEY-VCH Verlag GmbH *&* Co. KGaA, Weinheim.

Recently, silver nanocubes (AgNCs) have been functionalized with various ligands to enhance detection sensitivity for analytes of interest. An example is the modification of AgNCs with 4-aminothiophenol (4-ATP), which undergoes a specific chemical transformation when exposed to nitrite. The thiol groups of 4-ATP bind to the AgNCs via Ag–S bonds, while the amino group interacts with nitrite, forming the dimer 4,4′-dimercaptoazobenzene (DMAB). This reaction creates azo bonds that produce a distinct SERS signal for nitrite detection. The application of these AgNCs has been demonstrated by incorporating them into face masks designed to collect and analyze nitrite in exhaled breath condensation. The face masks feature hydrophilic squares that capture droplets from exhaled breath, enriching the analytes and AgNCs for in situ SERS analysis. This setup, shown in Figure 2a, enables real-time detection of nitrite in breath samples.^64^

In a similar strategy, shown in Figure 2b, AgNCs have been functionalized with thiophenol ligands, such as 4-mercaptopyridine (4-MPY), 4-mercaptobenzoic acid (4-MBA), and 4-ATP. These functional groups enhance the breathalyzer’s detection capabilities by facilitating strong interactions with VOCs from breath through hydrogen bonding, ion–dipole interactions, and π–π interactions. The custom SERS breathalyzer is a hand-held device designed for rapid and noninvasive mass screening of COVID-19. It consists of a SERS sensor nested within a single-use breath chamber, facilitating the safe collection of breath samples. From breath collection to result, the process takes less than 5 min, reportedly achieving over 95% sensitivity and specificity and generating detailed breath profiles.^65^ AgNCs functionalized with 4-MBA have been employed in a SERS-based hydrogen bonding induction strategy to detect gaseous acetic acid. The 4-MBA ligand captures acetic acid molecules through hydrogen bond formation, inducing significant changes in the SERS signals. This method allows for acetic acid detection at ppb levels. To implement this detection system, functionalized AgNCs were deposited onto a gold nanofilm-coated silicon wafer to create a sensing chip, enabling precise and sensitive SERS analysis of acetic acid.^66^ Another approach involves dendritic Ag nanocrystals, inspired by the structural features of moth antennae, which create numerous cavity traps. These cavities increase the interaction time between gaseous molecules and the sensor surface, significantly enhancing adsorption and detection sensitivity. When combined with a nucleophilic addition reaction between aldehydes and bound 4-ATP, this system allows for the sensitive detection of aldehydes as biomarkers for lung cancer. This SERS sensor was reported to detect aldehydes such as glyoxal, glutaraldehyde, benzaldehyde (BZA), and phenylacetaldehyde at ppb concentrations, ranging from 2.0 ppb to 20.0 ppm, with its performance unaffected by humidity.^67^

Recent advances in SERS have exploited the synergistic properties of metal nanoparticles and MOFs to create highly sensitive sensors for gaseous biomarkers, particularly aldehydes and ketones relevant to cancer diagnostics. A notable approach involves encapsulating silver nanoparticles (AgNPs) with a thin zeolitic imidazolate framework (ZIF-67) shell, which is about 10 ± 5 nm thick. This Ag@ZIF-67 complex is further functionalized with 4-ATP to selectively absorb aldehydes and ketones via the Schiff-base reaction, forming a SERS gas sensor. The dried composite powder is loaded into glass capillaries to detect volatile compounds in breath samples, providing a noninvasive method for cancer diagnostics.^68^ In a related approach, AgNPs coated with a ZIF-67 layer and graphitic carbon nitride (g-C_3_N_4_) are used to detect aldehydes in exhaled breath, specifically for lung cancer biomarkers. The system incorporates a multifunctional solid phase extraction (SPE) membrane that concentrates the analytes, while the g-C_3_N_4_ prolongs interaction time with the substrate, achieving an ultrasensitive limit of detection (LOD) for aldehydes at 1.35 nM.^69^ AgNP@ZIF nanoparticles, where AgNPs are coated with a ZIF shell of varying thickness (20, 30, and 50 nm) to form a plasmonic metal–organic framework (MOF) nanoparticle film, which serves as a highly sensitive sensor, have been designed for the detection of gaseous aldehydes, with a particular focus on glutaraldehyde, a well-known tumor biomarker found in the exhalation of lung cancer and gastric cancer patients. The sensor was reported to have a low detection limit for glutaraldehyde, reaching concentrations as low as 10^–8^ M.^70^

Another approach features flexible porous films from Ag nanowires (AgNWs) coated with ZIF-8 to prepare paper-based plasmonic MOF nanowire films. These materials enable the SERS detection of gaseous aldehydes and VOCs, including biomarkers such as BZA for colorectal cancer, with a reported 93.7% accuracy through an artificial neural network (ANN) model.^71^ A similar AgNW@ZIF-8 system has been applied for the early stage diagnosis of oral cancer, detecting methanethiol, a critical tumor biomarker, in simulated exhaled breath with an ANN model with reported 99% accuracy and an area under the curve of 0.996.^72^ In another design, poly(vinylidene fluoride) (PVDF) substrates coated with AgNPs, ZnO nanowires, and ZIF-8 layers have been developed to detect H_2_S gas in human breath. This system’s co-confinement of hotspots and analytes was shown to have high sensitivity, achieving a LOD of 10^–10^ v/v, useful for monitoring health and lifestyle impacts through breath analysis.^73^ In a similar approach, Ag nanowires coated with ZIF-67 or Co–Ni layered double hydroxides (LDH) have shown exceptional performance in detecting *p*-ethylbenzaldehyde (EBZA), a biomarker for lung cancer. The hollow structure of LDH nanocages enhances gas transfer, resulting in a remarkably low reported LOD of 1.9 ppb for EBZA, which significantly outperforms Ag@ZIF-67 and bare Ag nanowires. Gaseous aldehydes were collected using a functional device with the SERS sensor positioned in a homemade reaction chamber, shown in Figure 2c.^74^ Further advancements include the development of Ag nanoparticles coated with ternary FeCoNi-LDH nanocages for detecting a broader range of aldehyde VOCs, including BZA, formaldehyde, methylglyoxal, salicylaldehyde, furfural, and decanal. Specifically, the system demonstrated a LOD of 10 ppb for BZA. The Ag/Fe_0.07_(CoNi)_0.93_-LDH composite, enhanced by Fe^3+^ doping, increased the surface area and optimized the pore structure, allowing for superior adsorption of gas molecules. This modification also reduced the energy bandgap, enhancing excitonic transitions and charge transfer between the substrate and target molecules. Additionally, the substrate was functionalized with 4-ATP to facilitate selective adsorption of BZA. The classification of different aldehyde VOCs was performed using PCA, enabling discrimination between compounds. This method underscores the material’s high sensitivity and selectivity for aldehyde detection, particularly in cancer diagnostics.^75^

In another study, spherical AgNPs were combined with graphene and zinc oxide nanorods (ZnO NRs) to create a functionalized substrate. The ZnO NRs were first covered with graphene, and then, AgNPs were deposited on the surface. In this configuration, ZnO functions as an n-type thermoelectric semiconductor material. It generates a thermoelectric potential when a temperature gradient is applied, which modulates the electronic properties of the material adsorbed on its surface. This modulation was coupled with the presence of graphene, which has the potential for chemical enhancement in SERS, and the AgNPs, which enhance the electromagnetic field strength, significantly amplifying the SERS signals. The combined effects of ZnO’s thermoelectric properties, graphene’s chemical enhancement capabilities, and AgNPs’ electromagnetic field enhancement result in a highly sensitive SERS substrate. The SERS materials detected polystyrene microplastics and the SARS-CoV-2 spike protein (S protein).^76^

Another strategy explored a new and scalable substrate design featuring a particle-in-microporous–nanoporous structure composed of silver and silicon layers (Ag/Si/Ag). The Si porous micropyramid (Si PMDP) structure, enhanced with Ag nanoparticles and a Ag nanofilm, is designed to capture and concentrate more light through increased optical paths and light absorption caused by continuous round-trip reflection of light between the sidewalls of the adjacent Si PMDPs and the hierarchical nanoholes on the surface. The system is designed to detect gaseous aldehydes, such as EBZA, a biomarker for early lung cancer, through a breath bag with a detection limit of 0.1 ppb. The breath sampling was done using a medical breath bag with a small Ag/Si/Ag PMPD SERS chip that captures and analyzes the exhaled gases, shown in Figure 2d.^77^ The same group has further explored the concept of light trapping for the detection of H_2_S gas in exhaled breath with a substrate composed of textured silicon micropyramid/Al_2_O_3_ quasi-nanocavity/Ag nanoparticles/Au nanoshell (T-Si/Al_2_O_3_/Ag/Au), demonstrating excellent light-trapping effects, intensive multimode hotspots, and gaseous molecule capture capabilities. The substrate was shown to detect H_2_S at very low concentrations (0.1 ppb) using premodified reporter molecules like hexadecyl trimethylammonium bromide (CTAB), 4-nitrothiophenol (4-NTP), and acetamidobenzenesulfonyl azide (4-AA), which react with H_2_S to produce specific Raman spectral changes, which are then analyzed to identify and quantify the presence of the analyte. Using multiple reporters enhances the precision of detection, reducing interference and improving reliability.^78^

The use of bimetallic nanocubes (Au@Ag@Au NCs) and gold–silver core–shell nanocubes (Au@Ag NCs) has also been studied for gas sensing, specifically for detecting VOCs such as aromatics, aldehydes, ketones, and H_2_S. The system presents three detection units: one detects aromatic compounds through physisorption, utilizing their intrinsic strong SERS signals; another detects aldehydes and ketones via linker-mediated chemisorption, using the SERS signals of the chemical products formed with 2,4-dinitrophenylhydrazine (DNPH); and the third one detects H_2_S by the decreased SERS signal of a prelabeled Raman reporter due to the formation of Ag–S bonds. The sensor demonstrated the ability to simultaneously identify nine different volatile compounds with ultrahigh sensitivity at ppb levels with high selectivity. The sensor’s performance was evaluated in indoor environmental pollution monitoring and exhalation disease diagnosis. The choice of metal affects the sensor’s sensitivity and the type of gas it can detect. For instance, forming Ag–S bonds is crucial for detecting H_2_S. The sensor was reported to demonstrate high sensitivity (ppb level), selectivity, and robustness.^30^

### Gold-Based Sensors

Gold-based nanomaterials are excellent SERS candidates due to their plasmonic properties and unmatched chemical stability. Gold nanostructures, such as nanoparticles, nanorods, and nanoshells, can support LSPR, significantly amplifying the Raman signals from target molecules. This makes Au nanomaterials highly effective in SERS applications, particularly in environments where long-term stability is crucial. In addition to their plasmonic capabilities, gold nanomaterials offer superior resistance to oxidation and corrosion, making them highly reliable for long-term use in sensing platforms. Although gold is more expensive than silver, its stability and tunable surface chemistry have made it a preferred material for SERS substrates, particularly in applications requiring reproducibility and durability.

Gold superparticles (GSPs), composed of aggregated gold nanoparticles coated with a ZIF-8 MOF layer, have been utilized for lung cancer detection using gold as the main nanoparticle. The MOF layer slows the flow of gaseous aldehydes, facilitating adsorption onto the SERS-active GSPs and enhancing Schiff base reactions with 4-ATP. This system detects aldehydes at ppb concentrations, providing high selectivity.^79^ Further enhancements involve using hollow ZIF-8 coatings on GSPs, reducing the detection limit by an order of magnitude and improving mass transfer efficiency, making it more reliable for detecting biomarkers in exhaled breath. The hollow structure enhances the molecule permeation flux, reduces the adsorption of molecules in the MOF pores, and improves the mass transfer capacity. The hollow MOF can also effectively exclude interfering molecules, leading to a lower detection limit and more stable SERS signals, making it more reliable for detecting biomarkers in exhaled breath. The sensor is embedded into a breathing valve to create a mask-type sensor, as shown in Figure 2e. This setup allows the sensor to capture gas molecules from exhaled breath effectively. When a person wears the mask for about 20 min, the gas molecules interact with the sensor, which enhances the chemical reaction efficiency between the target molecules and the modified molecules on the GSPs, leading to stronger Raman signals.^80^ Similarly, mesoporous gold (MesoAu) coated with a hollow ZIF-8 layer exhibits remarkable sensitivity for detecting BZA, achieving a detection limit of 0.32 ppb. The interconnected mesopores and active sites of MesoAu enhance analyte diffusion, making this system highly effective for cancer biomarker detection.^81^

A SERS sensor has been used for the detection of hydrogen cyanide (HCN) gas, which is a potential biomarker for *Pseudomonas aeruginosa* lung colonization in cystic fibrosis (CF) patients. The researchers utilized gold-coated silicon nanopillars to create SERS substrates that enhance the Raman signal, allowing cyanide detection at very low concentrations. The experimental setup involved exposing the SERS substrates to HCN gas and measuring the Raman spectra. The detection limit was reported between 1.8 and 18 ppb. The study highlighted the potential of this SERS-based method for early detection of bacterial colonization in CF patients’ breath, which could reduce the need for invasive diagnostic techniques.^82^ The same group tested the sensor in the breath of children with CF. The results showed that while the method has potential, there were issues with background signals and false positives. The study suggests that a more careful selection of control subjects and a more extended study period are needed for more conclusive results.^83^

The integration of gold nanoparticles (AuNPs) with reduced graphene oxide (RGO) presents a promising approach for enhancing the detection of VOCs in breath analysis, particularly for diagnosing gastric cancer. RGO acts as a stabilizing agent. Additionally, it adsorbs and enriches VOC biomarkers from breath samples, acting similarly to an SPME fiber. This setup has been applied to breath analysis aimed at diagnosing gastric cancer to distinguish between early gastric cancer and advanced gastric cancer. In the study, 14 VOC biomarkers were identified and detected from breath samples. These include isoprene, menthol, pivalic acid, acetone, tetradecane, 2-methylpentane, 3-methylpentane, hexane, 2,3-dimethylpentane, 2-methylhexane, dodecane, hexanol, phenyl acetate, and 2-methylhexane.^61^

The vapor generation paper-based thin-film microextraction (VG-PTFM) device, designed for selective detection of BZA in exhaled breath, offers a hybrid sensing platform combining gold nanorods (GNRs) and quantum dots (QDs) encapsulated within the MOF NU-901. This system integrates fluorescence and SERS detection utilizing fluorescence resonance energy transfer (FRET) to enhance sensitivity. The addition of BZA disrupts these assemblies due to Schiff base reactions, increasing fluorescence, and Raman signals. The device can distinguish lung cancer patients from healthy individuals through BZA detection, with the fluorescence response being visually observable at concentrations as low as 0.05 ppm.^84^

Titanium dioxide (TiO_2_) is a multifunctional platform that enhances plasmonic activity through its high surface energy and ability to modulate electron transfer. A highly adsorptive Au-TiO_2_ nanocomposite sensor has been developed to detect SARS-CoV-2 spike proteins in respiratory aerosols, integrated within a face mask embedded with a SERS chip. The incorporation of TiO_2_ in this nanocomposite plays a critical role by significantly enhancing the substrate’s surface energy, which improves the adsorption of respiratory aerosols. This increased adsorption substantially enhances the SERS signal intensity, making the detection process more sensitive and effective. The study reports a 47% increase in SERS signals compared to those of conventional Au nanoislands, successfully detecting SARS-CoV-2 spike proteins at a concentration of 100 pM in aerosols. Additionally, the face mask serves to preconcentrate low-volume respiratory aerosols and achieves 98% accuracy in quantifying SARS-CoV-2 lysates when combined with an autoencoder prediction model. This Au-TiO_2_ nanocomposite offers a label-free approach for detecting SARS-CoV-2 in respiratory aerosols.^85^ In a related study, a SERS sensing platform for early lung cancer diagnosis was developed, featuring a TiO_2_ nanochannel membrane (TiO_2_ NM) asymmetrically coated with AuNPs and functionalized with 4-ATP via Au–S bonds. This structured TiO_2_ NM creates uniform SERS hotspots that enhance Raman signal sensitivity. The platform detects gaseous aldehydes, such as BZA, by exploiting the Schiff base reaction with the pregrafted Raman-active probe molecule, 4-ATP. The addition of a ZIF-8 layer further enhances gas capture and adsorption. PCA and machine learning (ML) models were applied to discriminate between detected gases, offering advanced selectivity in SERS-based diagnostics.^86^ In another approach, titanium dioxide nanowires (TiO_2_ NWs) were decorated with AuNPs and assembled into a 3D mesh by using dielectrophoretic self-assembly to form the SERS substrate. This method creates a dense network of plasmonic hotspots, dramatically improving the Raman signal intensity and detection limits. The sensor, tested using 4-ATP as a model analyte, achieved detection limits as low as 10 ppb_v_ in gas and approximately 2.4 pM in liquid. In a proof-of-concept experiment, the sensor successfully differentiated between exhaled breath condensates from individuals with upper respiratory tract infections (URTI) and healthy individuals, identifying 80% of the URTI group spectra as infection-related, highlighting its potential for breath-based diagnostics.^87^

### Other Metal-Based SERS Sensors

Metals other than gold and silver have been used for SERS sensing applications, providing alternative strategies for signal amplification and sensing. A novel SERS substrate has been developed chiefly on the basis of the chemical enhancement mechanism, which relies on the charge transfer processes between adsorbed molecules and SERS substrate. A sponge-like Cu-doped NiO (NiO_*x*_/Cu)-SnO_2_ p–n semiconductor heterostructure (SnO_2_–NiO_*x*_/Cu) can convert the concentration of VOCs into optical, machine-readable barcodes. It comprises p-type NiO_*x*_/Cu and n-type SnO_2_, forming a type-II p–n junction. The significant Raman enhancement (EF = 1.46 × 10^10^) is attributed to efficient charge separation facilitated by the p–n junction and the charge transfer resonance due to Cu doping. The porous structure further enriches probe molecules, magnifying the SERS signals. The SERS substrate can simultaneously detect multiple VOCs, including pyrene (PYR), 2-naphthalenethiol (2-NT), and EBZA, biomarkers for lung cancer. The detection limits for these VOCs are significantly lower than those obtained by other methods, such as GC-MS and fluorescence spectroscopy.^88^ A novel sensor based on hierarchical porous CuFeSe_2_/Au heterostructured nanospheres synthesized via a photoreduction method has been produced. These nanospheres were designed to detect lung cancer biomarkers, specifically aldehydes. The nanospheres contained a CuFeSe_2_ core with a Au shell. The porous nature of the nanospheres created cavity traps on their surface, increasing the reaction time of gaseous aldehydes with the sensor and thereby enhancing the detection sensitivity. 4-ATP was grafted onto the CuFeSe_2_/Au nanospheres. When aldehydes reacted with 4-ATP, a C=N bond formed, significantly altering the SERS signal and allowing for detection at a limit of 1.0 ppb. The nanospheres can be regenerated through photocatalytic degradation of adsorbed probes or biomolecules, making the sensor reusable and cost-effective.^89^

Perovskites have also been investigated for breath analysis. The system described by the authors as PervERS combined perovskites with an ultrathin Au coating. The PervERS system leveraged a steady-state-assisted band structure matching strategy, significantly enhancing vibronic coupling within the perovskite-molecule charge transfer complex. The gold coating broadened the resonance range, enabling effective Raman enhancement even for wide band gap molecules, thus allowing for sensitive and selective molecular detection. This resulted in substantial Raman enhancement and high photostability. This combination allowed for sensitive and selective molecular detection and was employed to detect aldehydes in exhaled breath for gastric cancer detection. The system employed 4-ATP functionalized Au@CsPbBr_3_ films to specifically recognize and capture gaseous aldehydes. The PervERS system was tested with breath samples from gastric cancer patients and healthy controls, showing discrimination between the two groups with an accuracy of over 81.09%. Moreover, perovskites exhibit versatile band structure tunability, which enables selective molecular detection across a broad range of compounds.^90^ The different types of nanomaterials as well as their target molecules are summarized in Table 1.

**Table 1:** SERS Substrates According to the Plasmonic Material Used and Their Performance

metal	nanomaterial	reported performance	application	ref
silver	AgNCs	10^–10^ M	breath nitrite detection	(64)
	AgNCs	sensitivity of 96.2% and specificity of 99.9% for COVID positive or COVID negative patients	COVID-19 breathalyzer	(65)
	AgNCs	60 ppb	gaseous acetic acid detection	(66)
	Dendritic Ag nanocrystals	2.0 ppb to 20 ppm	aldehyde detection	(67)
	AgNPs encapsulated with ZIF-67	3 ppb	aldehyde and ketone detection	(68)
	AgNPs@ZIF-67 with g-C_3_N_4_	1.35 nM	aldehyde detection	(69)
	AgNP@ZIF films	10^–8^ M	aldehyde detection	(70)
	AgNWs@ZIF-8	10^–8^ M	aldehyde detection	(71)
	AgNWs@ZIF-8	10^–7^ M	methanethiol detection	(72)
	PVDF substrates with AgNP/ZnO nanowires/ZIF-8 coatings	10^–10^ v/v	H_2_S gas detection	(73)
	Ag nanowires with ZIF-67 or Co–Ni LDH	1.9 ppb	aldehyde detection	(74)
	Ag NPs coated with FeCoNi-LDH	10 ppb	aldehyde detection	(75)
	AgNPs combined with graphene and ZnO nanorods	4 × 10^–8^ M	SARS-CoV-2 S protein detection	(76)
	Ag/Si/Ag systems with micropyramid silicon	0.1 ppb	aldehyde detection	(77)
	T-Si/Al_2_O_3_/Ag/Au substrates	0.1 ppb	H_2_S gas detection	(78)
	Au@Ag@Au NCs and Au@Ag NCs	ppb levels	aldehyde, ketone, and H_2_S gas detection	(30)
gold	GSPs with ZIF-8 MOF coatings	10 ppb	aldehyde detection	(79)
	Hollow ZIF-8 layers on GSPs embedded into a breathing valve to create a mask-type sensor	7.7 ppb	aldehyde detection	(80)
	MesoAu coated with hollow ZIF-8	0.32 ppb	aldehyde detection	(81)
	Au-coated silicon nanopillars	18 ppb	hydrogen cyanide detection	(82, 83)
	AuNPs with reduced graphene	specificity of >92% and sensitivity of 83% for diagnosis and stage of GC	detection of 14 VOC biomarkers	(61)
	VG-PTFM system integrating Au nanorods and quantum dots encapsulated within the MOF NU-901	0.1 ppb	aldehyde detection	(84)
	Au-TiO_2_ nanocomposite	100 pM	SARS-CoV-2 spike protein detection	(85)
	TiO_2_NM-AuNP with a ZIF-8 layer	0.19 ppb	aldehyde detection	(86)
	AuNPs on TiO_2_ nanowires for breath condensate differentiation	2.4 pM for 4-ATP, diagnostic classification of infected samples	4-ATP and upper respiratory infection diagnostics	(87)
other metals	sponge-like Cu-doped NiOx/Cu-SnO_2_ heterostructure	ppb level	PYR, 2-NT, and aldehyde detection	(88)
	CuFeSe_2_/Au nanospheres	1 ppb	aldehyde detection	(89)
	perovskite-based system Au@CsPbBr_3_	3.29 × 10^–9^ M	aldehyde detection	(90)

### Nanoparticle Modifications

Typically, colloidal nanoparticles without surface stabilizers are favored for SERS applications because capping ligands form a passivated surface, which can impede interaction with target molecules.^91^ Direct contact between the nanomaterial and the target molecule enhances the Raman signal and improves the detection sensitivity. Supporting these nanoparticles on various matrices, such as reduced graphene oxide, is beneficial to prevent excessive agglomeration and the loss of plasmonic properties.^61^

**Figure 3:**
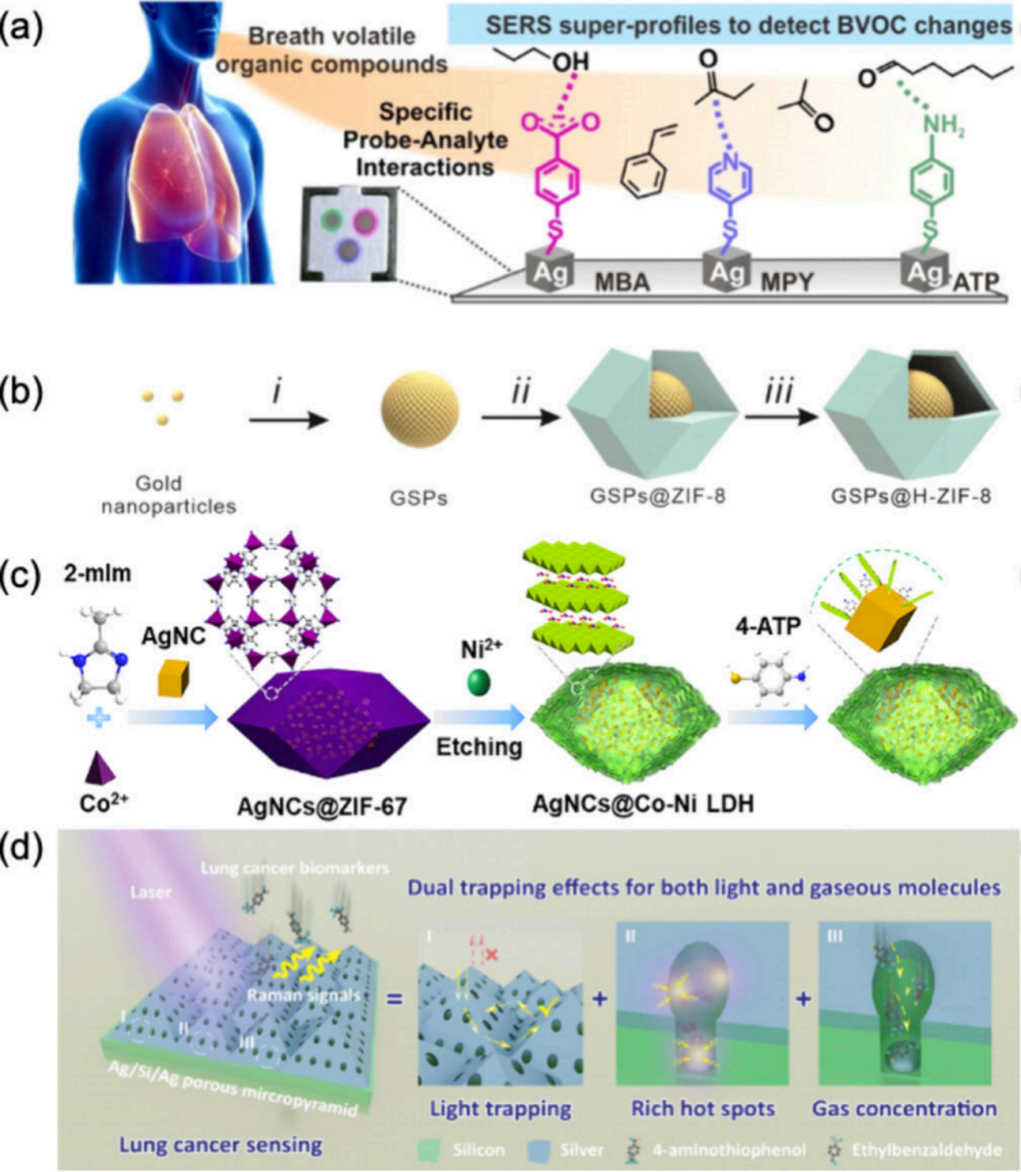
(a) Illustration of probe–analyte interactions in breath analysis for VOC detection. Breath samples are analyzed using various molecular probes attached to a silver substrate. Each probe is designed to interact with specific volatile compounds, facilitating selective detection. Adapted with permission from ref (65). Copyright 2022 American Chemical Society. (b) Schematic representation of the synthesis process for GSPs@H-ZIF-8 composite material from gold nanoparticles. Adapted with permission from ref (80). Copyright 2022 WILEY-VCH Verlag GmbH & Co. KGaA, Weinheim. (c) Synthesis process of AgNCs@Co–Ni LDH composite material through etching and functionalization steps. Adapted with permission from ref (94). Copyright 2021 American Chemical Society. (d) Enhanced lung cancer detection using dual trapping effects in Ag/Si/Ag porous micropyramids: light trapping, hot spots, and gas concentration. Adapted with permission from ref (77). Copyright 2023 WILEY-VCH Verlag GmbH & Co. KGaA, Weinheim.

However, surface functionalization of the plasmonic particles may also enhance their sensitivity or selectivity and have a positive impact in detecting gaseous analytes. A notable example is the modification with 4-ATP. This molecule forms strong bonds with nanoparticle surfaces through its thiol (−SH) groups, while its amine (−NH_2_) group remains available to capture target biomarkers such as aldehydes via the Schiff base reaction. The Schiff base reaction involves condensation of the primary amine group of 4-ATP with the aldehyde group of the target molecule, forming an imine (C=N) bond. This reaction allows the identification and quantification of different types of aldehydes. 4-MBA or 4-MPY functionalized onto the nanoparticles surface can similarly interact with VOCs via hydrogen bonding or ion–dipole interactions to bring the gaseous analytes close to the plasmonic surface,^65^ as shown in Figure 3a.

Various modifications and combinations have been applied to plasmonic nanoparticles to enhance the sensitivity and selectivity of the plasmonic and SERS-based sensors for breath analysis. These improvements primarily focused on increasing the contact time between VOCs and nanomaterials, thereby improving the detection capabilities. One notable approach involved using MOFs, which are coordination networks with organic ligands containing potential voids.^92^ MOFs (Figure 3b) have been employed to coat various nanomaterials, enhancing their ability to detect VOCs.^93^ The porous structure of MOFs helps to trap and concentrate VOCs, allowing for extended exposure to the nanomaterial surface. This increased contact time can significantly enhance the sensitivity of techniques like SERS, as the longer VOCs remain in proximity to the nanomaterial, the higher the probability of stronger signals and better detection. A similar strategy has been implemented using LDHs to improve the adsorption and capture of gaseous molecules.^94^ LDHs are alternating layers with an outer layer of positively charged metal hydroxides and an inner layer containing negatively charged anions and water. This structure provides a high surface area and numerous active sites for interaction with gaseous molecules (Figure 3c). The layered composition of LDHs offers advantages like those of MOFs, allowing for enhanced gas molecule adsorption and interaction.

In addition to trapping molecules in the vicinity of the nanoparticles, it is also possible to trap light. Light trapping involves structuring nanomaterials to maximize the interaction of light with the substrate^95,77^ (Figure 3d). The local electromagnetic field is significantly intensified by improving the light confinement and extending the path length of the incident light within the nanostructured material. This intensified field results in a substantial amplification of the intrinsic Raman signal, further enhancing the sensitivity and effectiveness of SERS. The combination of SERS with other amplifying techniques allows the increased interaction of the laser with the sample; for example, cavity-enhanced Raman spectroscopy (CERS) has been applied for VOC sensing.^96^ The combination of SERS and fluorescence as a dual-mode sensing strategy has also been studied, allowing the visual observation of VOCs and providing an alternative in gas detection.^84^ In this sense, the fabrication of the SERS substrate has been proven to be crucial, and different methodologies have been applied recently. The most prominent modification strategies are listed in Table 2.

**Table 2:** SERS Substrate Modifications for Specialized Applications

modification	application	refs
functionalization with 4-ATP, 4-MBA, 4-MPY, 4-NTP, or DNPH	detection of different molecules as aldehydes, ketones, acetic acid, and H_2_S gas	(30, 64−71, 73−75, 77−81, 84, 86, 89, 90)
MOF and LDH coatings for prolonged VOC interaction and enhanced trapping	enhancing VOC sensitivity via prolonged contact and detection	(68−73, 79, 80, 84, 86, 93, 94)
light-trapping designs intensifying Raman signals	amplified gas sensing and detection	(77, 78, 96)

### Microfluidic Integration for VOC SERS Analysis

Microfluidics provides significant advantages for gas analysis by enabling the manipulation of small fluid volumes within a larger system without the need for costly microfabrication techniques. This approach not only simplifies the system but also reduces its overall cost.

**Figure 4:**
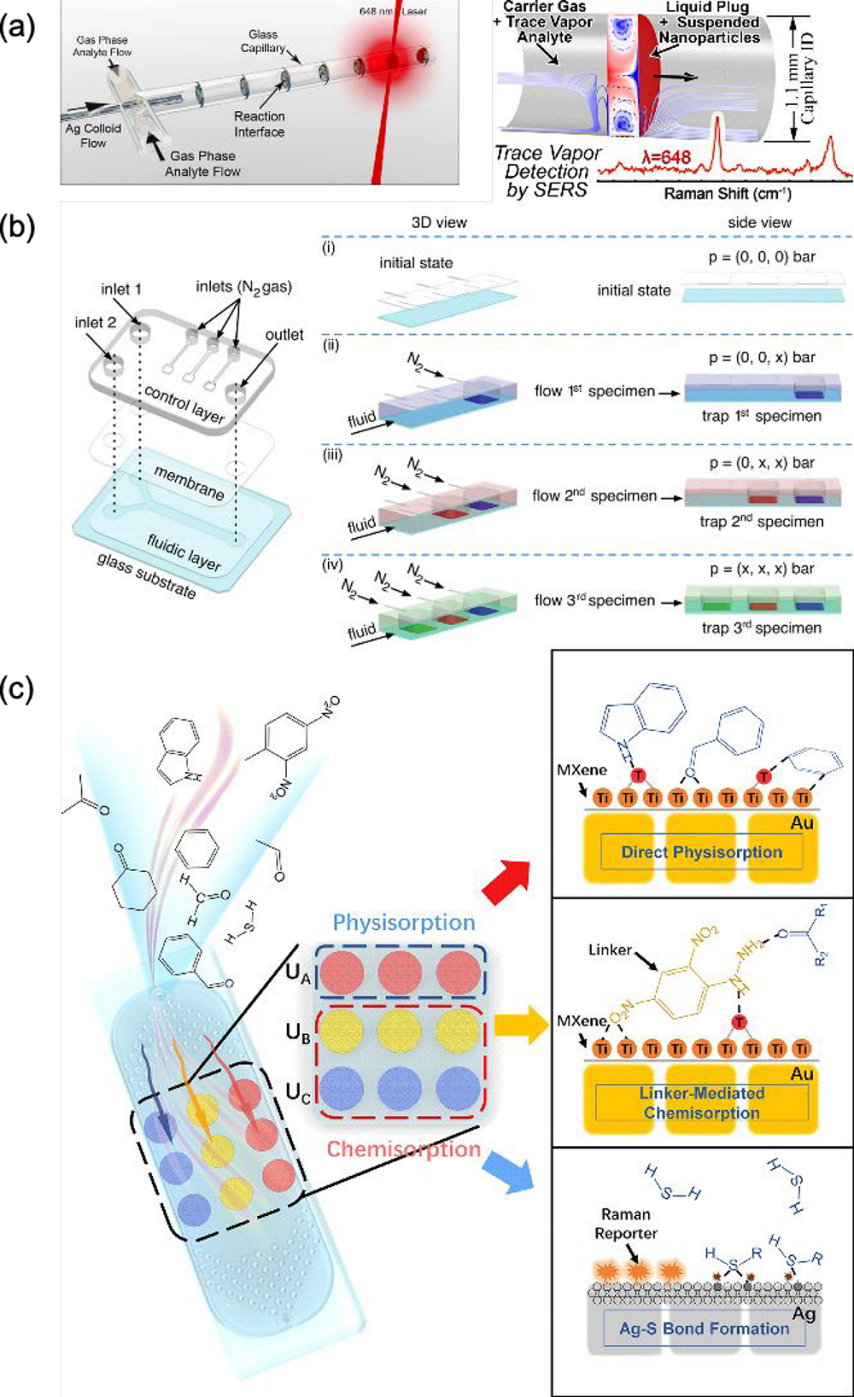
(a) Left: Illustration of a glass capillary setup where gas phase analyte and silver colloid flows intersect with a 648 nm laser used to analyze the system. Right: Schematic of a trace vapor detection system using SERS with a capillary setup, showing the interaction between carrier gas with trace vapor analyte and a liquid plug with suspended nanoparticles, resulting in enhanced Raman signal detection. Adapted with permission from ref (97). Copyright 2014 American Chemical Society. (b) Schematic of the operation of a microfluidic device for trapping multiple specimens. Left: The device consists of a control layer, a membrane, and a fluidic layer on a glass substrate, with inlets for fluid and nitrogen gas and an outlet. Right: The process involves four stages: (i) Initial state with no pressure applied. (ii) Flow of the first specimen into the device and trapping it by applying nitrogen gas. (iii) Introduction and trapping of the second specimen by adjusting the pressure. (iv) Flow and trapping of the third specimen, completing the multispecimen trapping process. Reproduced from ref (98). Available under a CC-BY 4.0 license. Copyright 2020 Sevim et al. (c) Schematic representation of the adsorption mechanisms on the MXene surfaces. Top: Direct physisorption of molecules onto MXene. Middle: Linker-mediated chemisorption involving a chemical linker between the molecule and the MXene. Bottom: Formation of Ag–S bonds with Raman reporters for enhanced detection. Adapted with permission from ref (30). Copyright 2022 American Chemical Society.

One effective microfluidic sensor in gas analysis employed a segmented flow technique using alternating liquid and gas regions. This configuration enhanced the interaction between gas-phase analytes and the liquid phase due to the high surface-to-volume ratio of the liquid plugs. By segmenting the gas into discrete liquid plugs, the system facilitated the rapid absorption of gaseous analytes into the liquid phase. This preconcentration process resulted in a higher concentration of analytes in the liquid phase compared to the gas phase. The microfluidic design supported continuous flow of gas and liquid phases, allowing for real-time monitoring and the detection of analytes. SERS-active silver nanoparticles were employed within the liquid plugs to generate intense Raman spectra (Figure 4a).^97^ Microfluidic devices also allow the simultaneous detection of multiple gases without cross-contamination at extremely low concentrations (down to 10^–14^ M). The configuration involves a double-layer microfluidic device made of polydimethylsiloxane (PDMS), with a control layer for pneumatic actuation and a fluidic layer for reagent flow. By manipulating the PDMS membrane through nitrogen gas pressure, specific areas of the fluidic layer can be isolated or exposed, allowing for sequential trapping and washing of different specimens. This technique ensures high sensitivity and prevents cross-contamination, as demonstrated by the controlled localization and detection of model analytes (e.g., crystal violet, 4-ATP, and rhodamine 6G) within a single channel (Figure 4b).^98^

Breath VOCs have been analyzed with an on-chip plasmonic sensor incorporating three different detection units, each designed to respond to specific gaseous molecules through either physisorption or chemisorption mechanisms. Integrating microfluidic and multiplex nanostructure components on one chip allows for programmable design, extensible functions, and on-chip signal amplification, which enhance sensor sensitivity, selectivity, and reproducibility. The sensor was fabricated using a microfluidic chip with a top PDMS layer containing a microcolumn premixer and a bottom PDMS layer with SERS detection areas. The detection areas were customized with a structure resembling Chinese Mahjong nine dots, each containing three dots for averaging results. The microfluidic chip design allowed for efficient gas enrichment and enhanced SERS signal readout. The microcolumn premixer ensured uniform gas distribution, and the microfluidic channels facilitated controlled gas flow over the detection units. Integrating microfluidics with SERS substrates resulted in high sensitivity, selectivity, and robustness, making the sensor suitable for complex sample analysis in real-world applications (Figure 4c).^30^

### Machine Learning Applied to Gas Phase SERS Analysis

Integrating ML into SERS is pivotal for enhancing its practical applications and leveraging its capabilities to achieve prompt and automated data interpretation.

The rapid and accurate processing of complex and highly dimensional Raman spectra presents a significant challenge for practical applications, particularly in complex scenarios such as breath analysis. Chemometric methods and, more recently, ML techniques have been employed to tackle this issue. Robust data interpretation models are essential for effective diagnostics and clinical monitoring. These algorithms reduce the spectral data’s dimensionality, removing complexity while preserving the essential information.

Among the various methods, PCA is the oldest and most common technique extensively used for data classification and characterization. PCA is an unsupervised linear unmixing method that reduces the dimensionality of large spectral data sets by transforming the data into a new space while preserving the most correlated features. This new space is spanned by the directions of highest covariance, known as “principal components” (PCs). Each PC is a linear combination of the original variables, in decreasing order of variability, in the data set. For instance, the first, PC1, captures the highest variance in the data set, while the following PCs (PC2, etc.) capture progressively less. This enables the distinction of differences/similarities between the data set components and the identification of data clusters.^99,100^ After identifying the defining features of the data set via PCA, data classification can be incorporated using linear discrimination analysis (LDA). As a classification technique, LDA provides class separability and can enhance the understanding of data distribution.^101^

PCA has been performed in several studies, mostly involving breath samples for cancer screening. Utilizing simulated and clinical samples, researchers successfully identified early stage (*n* = 55) and advanced stage (*n* = 89) gastric cancer patients as well as healthy individuals (*n* = 56). This investigation focused on AuNP-decorated RGO composites. Classification was performed using PC1 and PC2, resulting in a distinct clustering of the three groups: healthy, early stage, and advanced-stage, across both simulated and real breath data sets. The PCA reported an identification sensitivity of 83% and specificity of over 92%, indicating effective diagnosis and staging of gastric cancer.^61^ A similar methodology was adopted in other studies to validate the SERS performance for lung cancer,^74,77,79,80,81,84^ gastric cancer,^90^*Pseudomonas aeruginosa*,^83^ and diabetes.^102^ These studies collectively highlight the versatility of PCA in analyzing various conditions in breath samples.

Another dimensionality reduction technique commonly used in linear quantitative analysis is PLS-DA. PLS-DA as a supervised regression technique is based on the principles of PCA but requires prior labeling of the different groups in the training set. Unlike PCA, which describes the total variance within a data set, PLS-DA identifies features that maximize the intergroup distance, even if they do not contribute significantly to the total sample variance. Hence, it aims to explain the data set variances based on the interclass differences, which are calculated as latent variables (LVs) and serve as a new coordinate system on which samples are projected.^87,100^ However, PLS-DA should not be used casually, as it is prone to overfitting.^103^

**Figure 5:**
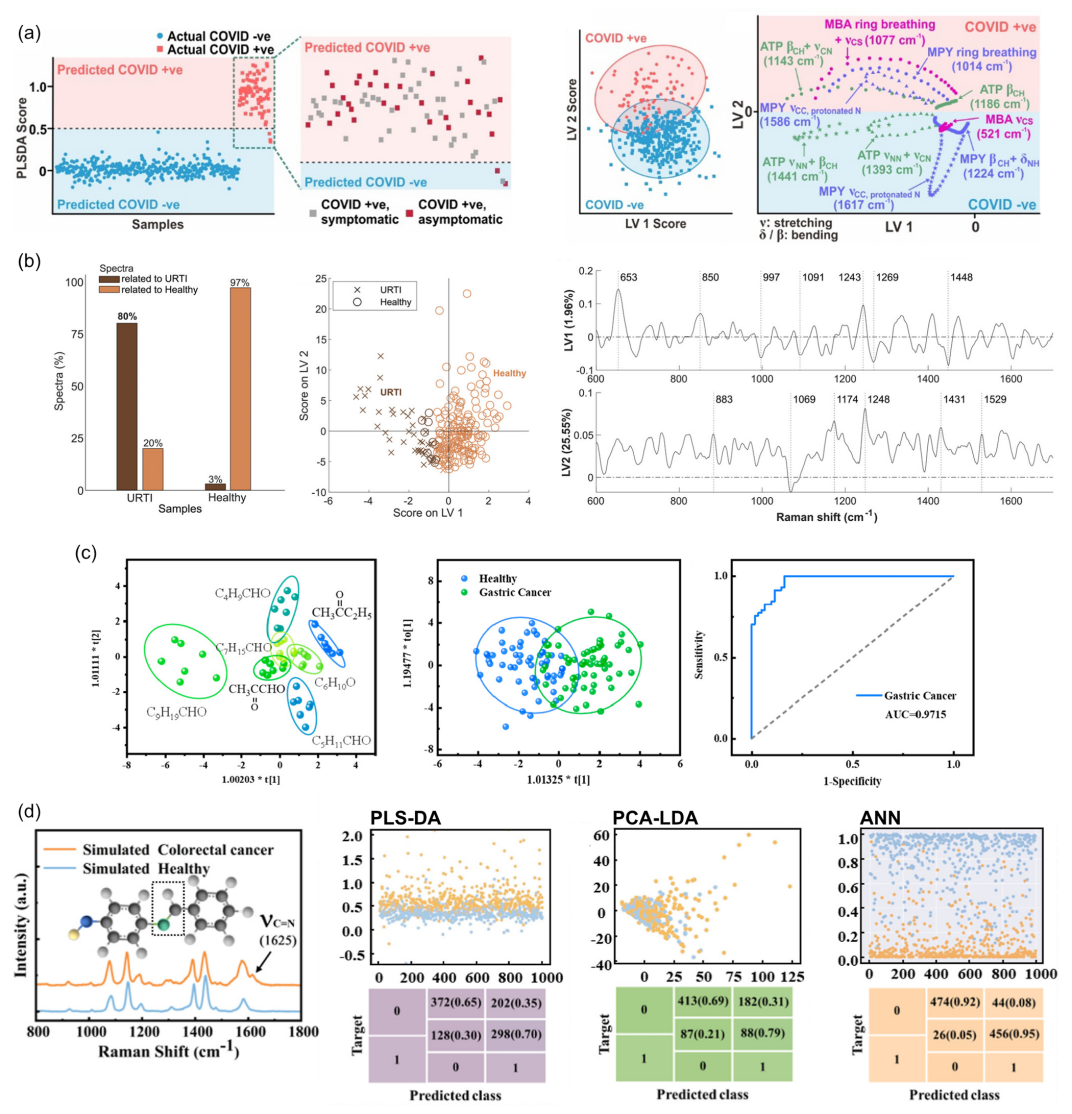
(a) From left to right: PLS-DA score-plot of COVID-positive and -negative individuals. (Inset: zoomed-in segment of COVID-positive individuals). Score-plots of LV1 and LV2. Loading plots are for LV1 and LV2. Adapted with permission from ref (65). Copyright 2022 American Chemical Society. (b) From left to right: OPLS-DA classification of “URTI” and “healthy” breath samples. Score-plots of LV1 and LV2. Calculated LVs. Adapted with permission from ref (87). Available under a CC-BY 4.0 license. Copyright 2024 Constantinou et al. (c) From left to right: OPLS-DA score-plot of seven different VOC biomarkers for gastric cancer. OPLS-DA score-plot of breath samples from gastric cancer patients and healthy individuals. Receiver operating characteristic (ROC) curve based on the OPLS-DA score plot of gastric cancer vs healthy profiles. Adapted with permission from ref (68). Copyright 2022 American Chemical Society. (d) From left to right: SERS spectra of simulated exhaled breath samples of colorectal cancer patients (orange color; labeled as “1”) and healthy subjects (blue color, labeled as “0”) and score-plots derived from PLS-DA, PCA-DA, and ANN classification. Adapted with permission from ref (71). Copyright 2024 American Chemical Society.

A study involving a large data set of over 500 participants was performed to distinguish COVID-19-related breath VOCs. This work used a hand-held SERS breathalyzer consisting of Ag nanocubes functionalized with molecular receptors to perform the breath analysis in under 5 min. The rapid analysis of the Raman data sets was achieved by incorporating PLS-DA to provide instantaneous results. Overall, the PLS-DA model reported an average classification sensitivity and specificity of 96.2% and 99.9%, respectively, when distinguishing positive and negative COVID breath samples. As shown in Figure 5a (left), the samples were clustered along LV2, with more positive scores for the COVID-positive samples and vice versa for the COVID-negative samples. The PLS-DA score-plot and loading plot showed the influence of LV2 on classifying COVID-positive from COVID-negative individuals (Figure 5a, middle to right). The high sensitivity levels of the breathalyzer highlight the robustness and accuracy of PLS-DA and its potential diagnostic utility.^65^

A modified version of PLS-DA, known as orthogonal partial least-squares (OPLS), has been employed in a recent study to identify URTI via breath samples. The OPLS-DA algorithm is a supervised model that orthogonalizes the calculated LVs. This component separation is useful in applications where understanding group differences is critical.^58^ A novel approach for identifying URTI was performed by using fully solution-processed TiO_2_ NWs decorated with plasmonic Au NPs. This work conducted a pilot study based on exhaled breath condensates collected from healthy and URTI-infected volunteers. Strict classification criteria were followed in this study, i.e., a sample was considered a member of a specific class only if the predicted probability for that class was greater than 50%. As shown in Figure 5b (left to middle), by employing OPLS-DA, 80% of the spectra collected from the sample of an infected individual were identified as being COVID-related, while 20% contained molecules unrelated to SARS-CoV-2. Some overlap was expected, as exhaled breath contains a variety of molecules unrelated to disease, which would be classified as “healthy” by the model. In the analysis, two LVs were used, with LV1 capturing 1.96% of the total data set variance that separated the URTI from healthy samples^87^ (Figure 5b, right). OPLS-DA was also performed on SERS-based breath analysis for gastric cancer screening using a tubular SERS sensor with a glass capillary loaded with Ag@ZIF-67. In this work, seven different biomarkers (including butanone, pentanal, hexanal, octanal, decanal, cyclohexanone, and methylglyoxal) were identified by GC-MS and applied further for the noninvasive diagnosis of gastric cancer using the tubular SERS sensor. Most of the data points were clustered independently and separated from each other in the OPLS-DA plot, demonstrating that the tubular SERS sensor can identify gastric cancer biomarkers (Figure 5c, left). The SERS sensor also detected exhaled breath samples from gastric cancer patients (*n* = 57) and healthy volunteers (*n* = 61). Using OPLS-DA, the SERS sensor successfully clustered the two groups, showing a small portion of overlap (Figure 5c, middle). The receiver operating characteristic (ROC) curve of 0.9715 reflects the excellent performance of the classifier (Figure 5c, right). This study reported high sensitivity and specificity of 91.23% and 88.52%, respectively, for gastric cancer diagnosis with the potential for medical diagnosis of other diseases.^68^

ANN algorithms have also been implemented for SERS analysis. The ANN was originally developed to mimic the learning process of the brain. Compared to the aforementioned methods, the ANN model can analyze very complex chemical data and nonlinear problems that demand high computational costs. The ANN involves three components: the input layer, the output layer, and the hidden layer, which consists of interconnected nodes (neurons). Therefore, each neuron (“hidden unit”) is composed of a regression equation that processes the input information into nonlinear output data. By increasing the number of neurons, nonlinear correlations can be treated.^104^

A recent study of breath-simulated spectra of colorectal cancer and healthy exhalations was performed to identify gaseous aldehydes (biomarkers of colon cancer) by comparing ANN with other chemometric tools, including PLS-DA, PCA, and PCA-LDA (Figure 5d). The ANNs used in the study consisted of 1 to 4 hidden layers, each with 1–50 interconnected nodes (neurons). The ANN algorithm was reported to achieved a high accuracy of 93.7% compared to the other methods that exhibited classification performance, demonstrating great potential in early stage diagnosis of colorectal cancer.^71^ Similar studies were performed from the same research group for oral cancer (99% accuracy)^72^ and gastric and lung cancer (89% accuracy),^70^ achieving high accuracy levels that illustrate the potential value of ANNs in breath-based diagnostics.

Overall, these methodologies highlight the significant role of ML techniques in enhancing SERS applications for rapid and accurate medical diagnostics through exhaled breath. As more studies are performed with larger patient pools and advanced sensors, the data analysis methodology will become crucial. Blindly following an analytical method can lead to overfit models, lending fake credibility to unsubstantiated results.

### Preconcentrators

One significant challenge in detecting VOCs in exhaled breath is their low concentration, since they appear in trace amounts. This limitation affects all detection methods, including SERS. To address this challenge, the concept of preconcentration has been explored. This is defined as the process of increasing the concentration of analyte in a solution prior to its detection.^105^ Thus, the sensors will be exposed to a markedly increased concentration than the ambient, and the detection threshold will be effectively reduced. Limited studies report the use of preconcentrators for SERS-based detection, so in this section, we expand the discussion to include other established and emerging techniques.

Preconcentration utilizes the physical phenomenon of adsorption of low concentration molecules in a gas mixture on solid surfaces and subsequently their desorption/release at higher concentration. Adsorption isotherms^106^ helped to understand the physics of adsorption and develop the preconcentration devices. The adsorption process efficiency depends on (a) the adsorption capacity, defined as highest mass or volume of gas that can be adsorbed under given conditions including pressure, temperature, and surface-gas interactions, and (b) the selectivity of adsorption, that is the relative adsorption of one constituent over others from a gas mixture.^105^

Preconcentration is widely used for gas-phase applications and is not limited to breath. Typically, a solid surface is coated with special types of materials, called adsorbents. These are categorized based on the main material:

(a) Carbon-based such as carbopack B, carbopack X,^107^ nanopowders,^108^ graphene and graphene oxide, carbon nanotubes, and more.^105^ This is the most popular category with advantages of large adsorption capacity, ease of preparation, low cost, availability, high chemical and mechanical stability, and low adsorption enthalpy.
(b) Polymer-based, which is also a very popular category and includes Tenax TA and PDMS.^109^ Polymers offer high surface area and high chemical stability and work for a variety of analytes.
(c) MOFs such as MOF-5, ZIF-7, and UiO-66 that have been used for sampling and preconcentration of acetone and isopropanol, which are established diabetes biomarkers.^110−112^ MOFs offer advantages like high surface area, catalytic activity, tailorable pore size, and structural diversity.^110,111,113^
(d) Oxide-based like zinc oxide used to detect benzene.^114^ There are a variety of metal oxides for detection of explosives and toxic vapors.^105^ Oxide-based sorbent materials offer specific binding interactions, ease of preparation, chemical and mechanical stability, and flexibility of dimensional growth.^105^

The adsorption efficiency of the sorbent directly affects the concentration factor, which is defined as the ratio of the concentration of analyte delivered to the detector just after desorption over the concentration just prior to the preconcentration step. The desorption step can be done isothermally with pressure difference or with an external energy source such as electron beam or electromagnetic radiation, but the most common method is thermal with resistive or other heating of the sorbent.^105^ Finally, the preconcentration step involves the flow of the gas mixture into the analyte. Ambient air or gases such as nitrogen or helium can be used as a carrier gas for forced convection, but passive diffusion has also been demonstrated.^115^

Various types of preconcentrators have been developed by the research community, and some have been commercialized. They all have the same task: to enrich the concentration of specific (trace) analytes in gas samples before the analysis/detection. This results in increasing the sensitivity and accuracy of gas detection analytical systems such as GC and MS where preconcentrators are used commercially and integrated upstream of the sensing system.^105,116,117^

Preconcentrators can be combined with different types of detectors. Apart from the gold standard GC-MS in laboratory settings, flame ionization detectors (FID), photo-ionization detectors (PID), thermal ionization detectors (TID), optical spectrometers,^105^ and even electronic noses^118,119^ have been explored. There is no universal preconcentrator design; therefore, the preconcentration strategy usually depends on the final application. The versatility of preconcentrators has allowed them to penetrate many analytical applications, including environmental and indoor air quality monitoring,^120^ security and defense for explosives detection,^121−124^ food industry, and, importantly, medical diagnostics such as exhaled breath.^112,118,125−127^ Here, we summarize various preconcentrators, based on the type of measurement, whether it is one-off sampling or continuous online.

SPME, developed in 1989, gained attention for VOC analysis in human breath starting in 1997, where it has been applied to the detection of compounds such as ethanol, acetone, and isoprene.^128^ The method integrates extraction, sampling, and concentration in a single one-off passive step using a fused silica fiber coated on the outside with a polymeric stationary phase, followed by desorption and analysis.^129^ The sampling step can be done by directly immersing the fiber in the gas or liquid sample or exposing it to the headspace above a liquid or a solid sample.^130^ This is the most common technique to isolate and concentrate a sample from a matrix and enable its subsequent analysis.^130^ Various coatings and combinations have been used since then.^131^ These include PDMS, polydimethylsiloxane/divinylbenzene (PDMS/DVB), carboxen/polydimethylsiloxane (Car/PDMS), DVB/Car/PDMS, polyacrylate (PA), and CARBOWAX poly(ethylene glycol) (PEG).^130^ SPME is used to extract and concentrate VOCs, pesticides, or drugs for environmental and food samples, biological fluids, and forensic science applications.^130^

Another one-off concentration method is via thermal desorption tubes (TDTs). They are made from either quartz or stainless-steel material and packed with sorbent material. These are macroscale tools and are fabricated with more traditional methods; thus they contain larger amounts of sorbent material and thus capacity but are slower during the desorption step. They are commonly found in analytical laboratories within the thermal desorption system (TD-GC-MS).^132,133^

Additionally, TDTs have been demonstrated to capture and retain VOCs from exhaled breath as it passes through the tube. The choice of sorbent material and how they are packed is crucial,^134^ from porous polymers to silica gel or carbon-based ones, among others, as different materials are suited for varying applications, allowing for selective targeting of specific VOCs. This adaptability makes TDTs a versatile method for VOC preconcentration in a wide range of analytical applications, including breath analysis.^135−137^

A similar and miniature version of the TDT is the needle trap device (NTD). It consists of a fine needle packed with sorbent materials with a side hole. These traps sample air through the needle, and the analyte adsorbs on the sorbent material that is then desorbed thermally. NTDs are usually used for one-time or short-term sampling, followed by thermal desorption into GC systems.^129^ NTDs can be filled with various types of sorbents, similarly to TDTs, including porous polymers (e.g., Tenax), carbon-based materials (e.g., CAR) or activated charcoal, DVB, and zeolites.^138−140^ NTDs have been demonstrated for exhaled breath sampling with or without a mask and showed good trapping capabilities.^139^

Other single sampling and concentration methods have been demonstrated via semipermeable membranes used to remove the solvents.^141,142^

Until 1979 most preconcentrator research relied on large instrumentation for analyte detection.^105^ In 1979 the first microscale GC was reported using miniature detection and marked the beginning of microscale preconcentrators.^143^ In 1989, the first online analysis was performed using a micro-preconcentrator tube with thermal desorption.^144^ A similar tube was used essentially as an online version of the NTD and served a dual functionality: preconcentration and injection with rapid thermal desorption.^145^

Since then, extensive research has been demonstrated on micro-preconcentrator designs for online concentration and integration with micro-gas chromatography systems. Instead of tubular shapes, the micro-preconcentrators have planar designs with microchannels fabricated with various shapes and dimensions and filled with all sorts of sorbent materials.^146^ The fabrication techniques take advantage of micro-electromechanical systems (MEMS) and complementary metal-oxide semiconductor (CMOS) technologies for silicon, metal, ceramic, and glass substates.^121,147−151^ Researchers have utilized micro- and nanosized features in the microchannels to increase the surface area and thus exposure of the analyte to the sorbent.^152^ Such channels include (a) microcavity designs, (b) parallel channel arrays, (c) tapered cavities, and (d) spiral designs.^147^ The push for miniaturization comes with challenges but also important advantages such as the possibility of (a) integration of microheaters into the system to rapidly desorb gases for analysis,^149,153,154^ (b) integration with the sensor in a compact and efficient manner for real time monitoring, and (c) increased energy efficiency for desorption due to smaller volume of material.

Moreover, the miniaturization opened the possibility of designing multiple stage preconcentrators. The first is the retention step, and the second is the injection step, which is smaller. In 1996 the first multistage preconcentration was demonstrated.^155^ In 1998, the first two-stage microtrap was shown to work in an online setting with retention and injection steps.^156^ These multistage preconcentrators were integrated with micro-GC systems with a microfabricated column that allowed for analyte selectivity using microsensors at the output.^157−159^

Selectivity can be achieved directly from the preconcentrator step. If the channel with the sorbent material is long enough, it can serve as a chromatographic column. The adsorbates will desorb and come out at different time points, allowing the sensor to detect one at a time.^160^

Thus, microfabricated planar preconcentrators are suitable for portable GC systems^161−165^ and hand-held devices, where size is essential, on-site environmental monitoring^124^ and hazard detection,^124^ and other chemical sensing applications.^166^

Significant efforts from the research community have developed this technology to a very advanced level. However, there is potential for further innovation and improvements. Specifically, a larger range of materials can be explored for sorbents, and gas mixtures instead of single analyte in carrier gas need to be tested. The selectivity of gas detection and analysis systems needs significant improvement, and this can partly come from the preconcentrator design itself. Finally, the detector integration with the preconcentrator is a territory for impactful innovation. The preconcentrators cannot deliver meaningful results on their own, and the detector of choice is crucial for successful real-life applications. Pairing a long-channel micro-preconcentrator with a detector that offers selectivity, such as SERS, can be a key innovation for miniaturized, fast, and accurate gas analysis systems.

## Conclusions

Breath analysis is here to stay as a noninvasive diagnostic methodology. It has seen increasing interest due to its potential to identify diseases based on VOCs emitted from within the body. SERS lends itself naturally to breath analysis, as it can detect VOCs reasonably well at trace concentrations, without being affected by the high water content of the sample, and can potentially distinguish pathologies, respiratory and otherwise. For example, SERS has demonstrated the ability to discern differences in the VOC profiles of early and advanced stages of gastric cancer, offering a route for early diagnosis. The significant advancements in SERS over the past years, yielding a plethora of novel plasmonic substrates, have opened up its promising potential for VOC detection, facilitating breath analysis.

However, the application of SERS in breath analysis faces its own set of challenges, including ensuring the reproducibility of measurements, enhancing the molecular selectivity, and managing the complexity of breath composition. Nonetheless, with ongoing developments in nanomaterials and advances in the control of plasmonic surfaces, SERS-based techniques hold promise for revolutionizing the way we perform noninvasive diagnostics. At the same time, the integration of SERS detection with preconcentrator systems and microfluidic sample handling will enhance the relevance and usability of the technique. Careful and responsible use of chemometric and ML methodologies needs to be performed to ensure consistent and meaningful interpretation of the measurements. Table 3 summarizes the different techniques mentioned that can help in advancing breath analysis in combination with SERS spectroscopy.

**Table 3:** Different Methodologies and Techniques That Can Be Used in Combination with SERS for Breath Analysis

preconcentrators	microfluidic systems	machine learning techniques
solid-phase microextraction (SPME): refs (128−131)	segmented flow microfluidics: alternating liquid–gas flow enhances analyte absorption into liquid plugs, enriching analytes for real-time SERS: ref (97)	dimensionality reduction and clustering: PCA is used to reduce spectral dimensionality and identify clusters, often as a precursor to supervised methods: refs (61, 99, 100).
thermal desorption tubes (TDTs): refs (132−137)	double-layer PDMS microfluidics: pneumatic actuation isolates channels, ensuring sequential trapping and preventing contamination: ref (98)	supervised classification: techniques like LDA and PLS-DA/OPLS-DA classify VOC clusters and groups, emphasizing class separability using regression-based and linear discriminant approaches: refs (58, 61, 65, 68, 87, 100, 101, 103)
needle trap devices (NTDs): refs (129, 138−140)	on-chip plasmonic sensor with premixer: integrates multiplexed SERS substrates for VOC detection with high sensitivity and selectivity: ref (30)	neural network-based models: ANNs and deep learning architectures analyze VOC data, enabling nonlinear analysis and multilayered processing for complex spectra: refs (70−72)
micro-preconcentrators: refs (147−165)		hybrid methods: combined approaches integrating unsupervised–supervised techniques or regression with neural learning to enhance classification: refs (61, 71, 74, 80, 101)
multistage preconcentrators: refs (155−159)		

Future efforts may focus on integrating portable SERS devices into clinical settings, increasing the diagnostic value of these platforms and developing selective detection protocols that can isolate specific VOC biomarkers with higher precision. As research in this field progresses, SERS may become integral to personalized health monitoring and disease detection, complementing GC-MS analysis, offering real-time analysis with minimal patient discomfort.

## Abbreviations

DNPH: 2,4-dinitrophenylhydrazine
2-NT: 2-naphthalenethiol
DMAB: 4,4′-dimercaptoazobenzene
4-ATP: 4-aminothiophenol
EBZA: 4-ethylbenzaldehyde
MBA: 4-mercaptobenzoic acid
MPY: 4-mercaptopyridine
4-NTP: 4-nitrothiophenol
4-AA: acetamidobenzenesulfonyl azide
ANN: artificial neural network
BZA: benzaldehyde
Car/PDMS: carboxen/polydimethylsiloxane
CERS: cavity-enhanced Raman spectroscopy
CF: cystic fibrosis
DA: discriminant analysis
DVB: divinylbenzene
FID: flame ionization detectors
GC: gas chromatography
GC-MS: gas chromatography–mass spectrometry
Au: gold
AuNP: gold nanoparticle
GNR: gold nanorod
GSP: gold superparticle
CTAB: hexadecyl trimethylammonium bromide
H-ZIF-8: hollow ZIF-8
IMS: ion mobility spectrometry
LV: latent variable
LDH: layered double hydroxide
LOD: limit of detection
LDA: linear discrimination analysis
LSPR: localized surface plasmon resonance
LC: lung cancer
ML: machine learning
MS: mass spectrometry
MesoAu: mesoporous gold
MOF: metal–organic framework
CMOS: metal-oxide semiconductor
MEMS: micro-electromechanical system
PMDP: porous micropyramid
NM: nanochannel membrane
NW: nanowire
NTD: needle trap device
OPLS: orthogonal partial least squares
PLS: partial least squares
ppb: parts-per-billion
PID: photoionization detector
PA: polyacrylate
PDMS: polydimethylsiloxane
PDMS/DVB: polydimethylsiloxane/divinylbenzene
PEG: poly(ethylene glycol)
PNIPAm: poly(*N*-isopropylacrylamide)
PVDF: poly(vinylidene fluoride)
PCA: principal component analysis
PC: principal component
PA: *Pseudomonas aeruginosa*
PYR: pyrene
QD: quantum dot
RGO: reduced graphene oxide
RH: relative humidity
SIFT-MS: selected-ion flow tube-mass spectrometry
Ag: silver
AgNC: silver nanocube
AgNP: silver nanoparticle
AgNW: silver nanowire
SPE: solid phase extraction
SPME: solid-phase microextraction
SERS: surface-enhanced raman scattering
TDT: thermal desorption tube
TID: thermal ionization detector
TOF-MS: time-of-flight mass spectrometry
TiO_2_: titanium dioxide
URTI: upper respiratory tract infection
VG-PTFM: vapor generation-paper-based thin-film microextraction
VOC: volatile organic compound
ZIF: zeolitic imidazolate framework
ZnO NR: zinc oxide nanorod

## Vocabulary Section

**Surface-Enhanced Raman Spectroscopy (SERS)**: analytical technique that significantly enhances the Raman scattering of molecules adsorbed on nanostructured metallic surfaces, enabling highly sensitive detection of molecular vibrations down to the single-molecule level.
**Nanomaterials**: materials with at least one dimension in the nanometer scale (1–100 nm), exhibiting unique physical, chemical, and mechanical properties that differ significantly from their bulk counterparts.
**Volatile Organic Compounds (VOCs)**: organic chemicals with high vapor pressure at room temperature, allowing them to easily evaporate into the air.
**Machine Learning**: subset of artificial intelligence that enables computers to learn patterns and make decisions or predictions without being explicitly programmed, improving their performance over time through the analysis of data.
**Preconcentrators**: devices or systems designed to enhance the detection of trace substances by collecting and concentrating low-abundance analytes from a sample before analysis.
**Microfluidics**: technology of manipulating and controlling fluids at the microscale, typically in channels with dimensions ranging from tens to hundreds of micrometers.
**Breath Sensors**: analytical devices designed to detect and quantify specific compounds in exhaled breath, providing noninvasive insights into health, disease, or environmental exposure.
